# E-cycling and health benefits: A systematic literature review with meta-analyses

**DOI:** 10.3389/fspor.2022.1031004

**Published:** 2022-10-19

**Authors:** Amund Riiser, Elling Bere, Lars Bo Andersen, Solveig Nordengen

**Affiliations:** ^1^Department of Sport, Food and Natural Sciences, Faculty of Education, Arts and Sports, Western Norway University of Applied Sciences, Sogndal, Norway; ^2^Department of Sport Science and Physical Education, University of Agder, Kristiansand, Norway; ^3^Department of Health and Inequalities, Norwegian Institute of Public Health, Oslo, Norway; ^4^Centre for Evaluation of Public Health Measures, Norwegian Institute of Public Health, Oslo, Norway

**Keywords:** active transportation, E-bicycle, health, fitness, meta-analysis

## Abstract

The objective of the present study is to review and meta-analyze the effect of E-cycling on health outcomes. We included longitudinal experimental and cohort studies investigating the effect of E-cycling on health outcomes. The studies were identified from the seven electronic databases: Web of Science, Scopus, Medline, Embase, PsycINFO, Cinahl and SportDiscus and risk of bias was assessed with the revised Cochrane Collaboration Risk of Bias Tool (RoB2). We performed meta-analysis with random effects models on outcomes presented in more than one study. Our study includes one randomized controlled trial, five quasi experimental trials and two longitudinal cohort studies. The trials included 214 subjects of whom 77 were included in control groups, and the cohort studies included 10,222 respondents at baseline. Maximal oxygen consumption and maximal power output were assessed in four and tree trials including 78 and 57 subjects, respectively. E-cycling increased maximal oxygen consumption and maximal power output with 0.48 SMD (95%CI 0.16–0.80) and 0.62 SMD (95%CI 0.24–0.99). One trial reported a decrease in 2-h post plasma glucoses from 5.53 ± 1.18 to 5.03 ± 0.91 mmol L^−1^ and one cohort study reported that obese respondents performed 0.21 times more trips on E-bike than respondents with normal weight. All the included studies had a high risk of bias due to flaws in randomization. However, the outcomes investigated in most studies showed that E-cycling can improve health.

## Introduction

Cycling is regarded a good form for physical activity and cycling conventional bikes has positively been related to health outcomes. In the two systematic reviews with meta-analyses of Nordengen et al. (1, 2), cyclists were associated with 16% risk reduction in cardio vascular disease (CVD) incidence and CVD mortality, and a 25% risk reduction of CVD risk factors as elevated blood pressure, overweight and obesity, low fitness, and unfavorable blood lipid profile.

We are in an electrically assisted bicycle (E-bike) revolution. Sales of E-bikes in Europe has increased from 0.5 million in 2009 to 3 million in 2019 (3), and they are steadily increasing to 4.5 million in 2021 (4). E-bikes differ from conventional bikes as they provide electrically powered assistance when riding. There are two different types of E-bikes; both where pedaling is required to get assistance and E-bikes that do not require pedaling (5). The present review will only refer to E-bikes as E-bikes which require the rider to pedal.

E-cycling compared to cycling is less intensive, however, the intensity is still within the intensity range for physical activity recommended by the WHO to obtain significant health benefits and mitigate health risks (6, 7). E-cycling could therefore help individuals meet physical activity recommendations. Several studies report that people cycle more and longer distances with an E-bike (6, 8). If E-cycling contributes significantly to the recommended 150 min per week with moderate physical activity, using an E-bike might therefore result in improved health. The E-bike might also shift mode of transport from car dependence (9), reducing man-made climate gas emissions.

A recent review and meta-analysis quantified the difference in acute physiological responses between E-cycling with electrical assistance, E-cycling without assistance, conventional cycling, and walking. Heart rate, oxygen consumption, and metabolic equivalents responses were lower when E-cycling compared to conventional cycling. However, E-cycling was associated with moderate to vigorous physical activity. E-cycling was also performed with a higher heart rate and oxygen uptake than walking and they concluded that E-cycling was associated with increased physiological responses that can confer health benefits (10). However, the impact of physiological and mental health arising from riding an E-bike is still inconclusive (3).

As demonstrated in several cross-sectional studies and acute experiments E-cycling has a great potential in improving health. However, few long-term studies have been conducted regarding the health effect of using E-bikes. Therefore, the objective of the present study is to review and meta-analyze the effect of E-bikes on health outcomes including intervention studies and longitudinal cohort studies.

## Methods

We performed a systematic review and meta-analysis according to the PRISMA 2020 guidelines (11). The research protocol for this systematic review with meta-analyses was registered at PROSPERO on 12 April 2022, with registration number CRD42022316485.

### Search strategy and selection criteria

#### Literature search

We selected the 17 studies identified by “Health benefits of electrically-assisted cycling: a systematic review” (12). In addition a university librarian systematically reproduced the search performed by Bourne et al. (12) and searched for studies published in the period from 2018 to 7th of March 2022. Peer reviewed publications in English were identified from the seven electronic databases: Web of Science, Scopus, Medline, Embase, PsycINFO, Cinahl and SportDiscus. The search consisted of the search terms “pedelec,” “E-bike,” “electrically assisted bicycle,” “electrically assisted cycle,” “electrically assisted bike,” “pedal-assist,” “electric bicycle,” “electric bike,” “electric cycle,” “electric mobility.” For full search strategy see Supplementary Table 1.

#### Inclusion criteria and the selection process

Two authors (SN and LBA) independently screened the 3,481 records on basis of title/abstract for eligibility and assessed 34 full text articles for eligibility. Discrepancies were resolved by discussion. We included experimental, quasi-experimental and cohort studies with a longitudinal design investigating the effect of E-cycling on outcomes related to health. Experimental studies were considered longitudinal if they investigated the effect of an intervention lasting more than 1 week and cohort studies were considered longitudinal if the cohorts were investigated more than once. Studies were eligible for inclusion if they included healthy participants ≥18 years of age and the electrically assisted bicycles had pedals and was operated by the individual, with assistance available from an electric motor. Studies examining outcomes related to cardiorespiratory fitness, like maximal oxygen consumption and maximal power output in an incremental trial, physiological outcomes like blood pressure and blood lipids, and questionnaire-based outcomes from validated questions/questionnaires aiming to assess mental and physical health were included. Studies comparing E-cycling to no control group, conventional cycling, passive transport, public transport, “business as usual” and walking were included. Field studies investigating the acute effect of E-cycling, observational studies, and studies on patient populations like heart disease and type 2 diabetes were excluded while overweight populations were included.

### Analysis

One authors (AR) extracted data from the included studies. Meta-analyses were performed when the same outcomes were reported in two or more studies and if the studies had a similar comparator (conventional bicycle was considered on type of comparator and passive travel group and no cycling control group was considered on type of comparator) regardless of length of the intervention, amount of cycling, or type of e-bike used in the intervention. In trials without a control group the change in the experimental group was compared to a hypothetical group with size and standard deviation equal to the experimental group and a change in the outcome of interest set to zero. These trials were meta-analyzed with trials with passive travel group and no cycling control group. The study by Hochmann et al. (13) is a randomized controlled trial (RCT) comparing the effects of E-cycling with conventional cycling. This is the only study with physiological outcomes and comparing it to conventional cycling. Thus, to include the results from this study in the meta-analysis, the results were compared with a hypothetical control group with size and standard deviation equal to the experimental group and change from pre to post of zero (the same procedure as we used with studies without a control group).

### Study quality assessment

The included studies were assessed using the five domains of the revised Cochrane Collaboration Risk of Bias Tool (RoB2) (14). The tool is developed for randomized controlled trials, but we used the same tool for all included studies. According to the tool criteria each domain was scored as low risk of bias, some concerns or high risk of bias. The domains in the tool are Risk of bias (1) arising from the randomization process, (2) arising from period and carryover effects, (3) due to deviations from the intended interventions, (4) due to missing outcome data, (5) in measurement of the outcome, (6) in selection of the reported result. Overall risk of bias for each study was determined by the highest risk of bias across all domains. Two authors (EB and LA) independently assessed the included studies and discrepancies in the assessment were resolved by discussion.

### Statistics

We performed meta-analysis with random effects models as we deemed the included studies to be heterogeneous with regards to interventions, study designs, and populations. We used Comprehensive Meta-Analysis (CMA) V3 (Biostat, Englewood, New Jersey, USA) to perform meta-analysis. Only one outcome per study was included in each meta-analysis and only the same outcomes from different studies were meta-analyzed. Effect estimates were presented as standardized mean difference (SMD) with 95% confidence intervals (CI) and in forest plots. Heterogeneity was reported as I^2^ and *p*-values. Significance level was set to *p* < 0.05.

## Results

### Study characteristics

**Figure 1:**
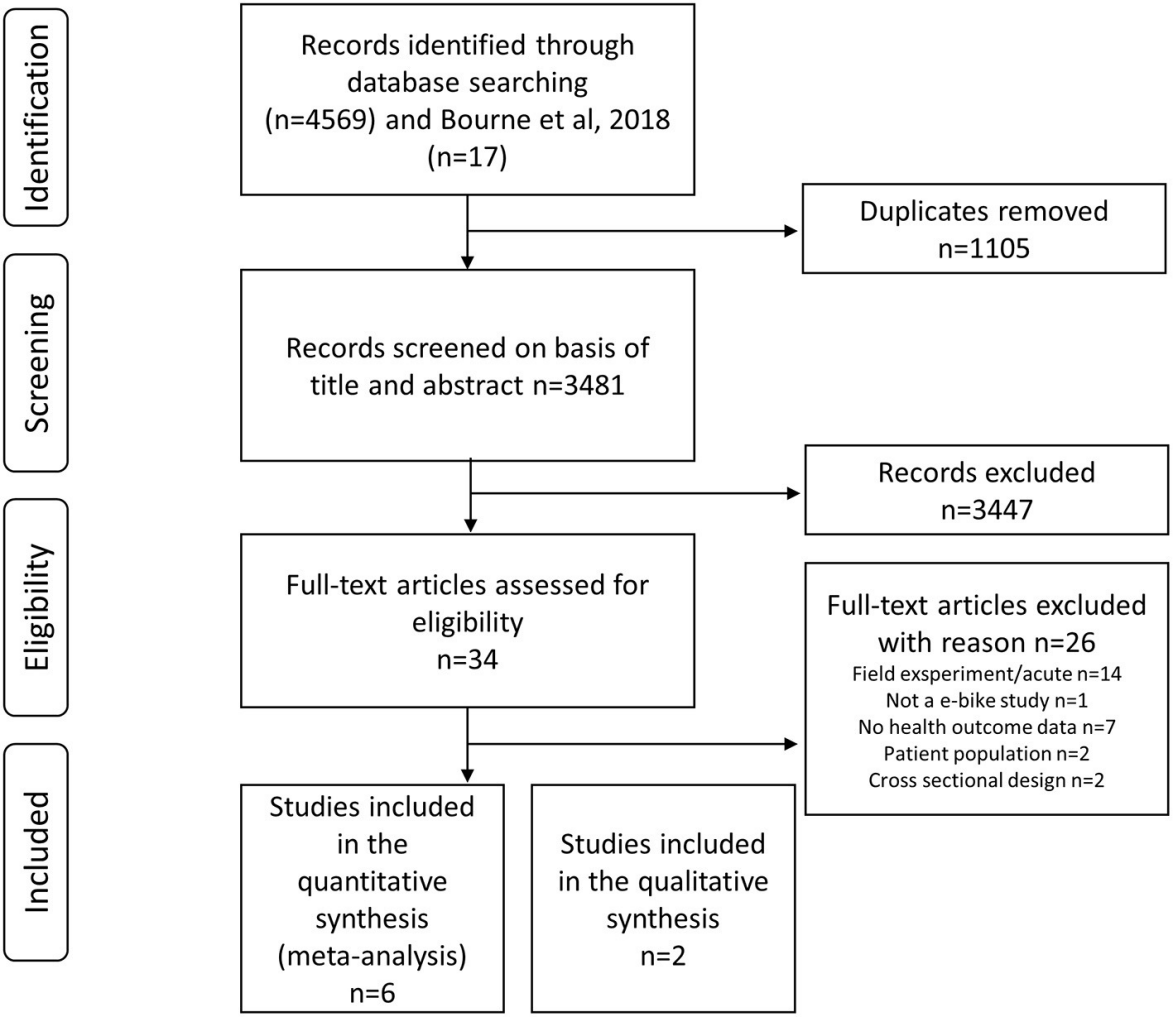
Flow chart of included studies as proposed by Preferred Reporting Items for Systematic Reviews and Meta-Analyses statement 2020.

The search identified 4,569 records (Web of Science 1328, Scopus 2533, Medline 221, Embase 239, PsycINFO 54, Cinahl 60, SportDiscus 134 records). After automatic elimination of duplicates, 3,481 records remained. Thirty four studies were selected for full-text eligibility assessment after screening of titles and abstracts (Figure 1).

In total eight studies were included in the systematic review (13, 15–21). We included one randomized controlled trial (13) and five quasi experimental trials, where one of the quasi experimental trials had a pseudo randomized control group (15), and the remaining four had no control group (16–19). We also included two longitudinal cohort studies (20, 21) investigating the association between E-cycling and health outcomes. The trials were performed in Switzerland, UK (x2), Norway, Belgium and USA and included 214 subjects of whom 77 subjects were included in control groups. Four trials investigated the effect of E-cycling on cardiorespiratory fitness, two the effect on body mass index (BMI), two the effect on blood pressure and two studies investigated the effect of E-cycling on mental health using the short form of the Global Health Questionnaire (GHQ12) (22) or the short form Health Survey (SF36) (23). One cohort study was from the Netherland and the other was a multicentre study from seven European cities. For characteristics of the included studies see Table 1.

**Table 1:** Characteristics of the included studies.

**Study**	**Study design**	**Duration**	**Participants, Gender, age (years)**	**Intervention/behavior of interest**	**Comparators**	**Outcomes**
Hochsmann et al. (13)	RCT, Switzerland	4 weeks	28 m/4 f	E-bike up to 250 W, active commute to work at a self-chosen speed on at least 3 days per week	Conventional bike	VO_2_-peak
			17 E-bike, 37 year (IQR 34, 45)			Max watt Body mass
						SBP
			15 Conventional bike (37 year) (IQR 38, 45)			
						DBP
						HR at rest
						BMI
			Overweight			
Leyland et al. (15)	Quasi-experimental, United Kingdom	8 weeks	100 (50–83 year), 38 E-bike, 26 non cyclist, 36 conventional bike	E-bike	Conventional bike, passive travel	Mental health
				Required to cycle at least three times a week for 30 mins		
						Physical health
			Older adults			
Page et al. (16)	Quasi-experimental, United Kingdom	Median 6 weeks, range 3–8 weeks	4 m/17 f, 21–55 yr	Multicomponent intervention including possibility to borrow E-bike free of charge	Passive travel	Physical health (self-report)
Lobben et al. (18)	Quasi-experimental, Norway	3–8 months	21 (baseline 7m/18f), 44 ± 7 year	Access to E-bike	No control group	VO_2_-max
de Geus et al. (17)	Quasi-experimental, Belgium	6 weeks	10 m, 10 f	Use E-bike at least 3 times per week to and from work 405 ± 156 km	4 weeks control period	VO_2_-peak
			45 yr ± 7/43 yr ± 6			Max watt
Peterman et al. (19)	Quasi-experimental, USA	4 weeks	14 f/6 m	Commute with E-bike at least 3 days per week for 40 min per day	No control group	BMI
			41.5 ± 11.5 yr			
						VO_2_max
						Max watt
						SBP
						DBP
Avila-Palencia et al. (20)	Observational Longitudinal study and cross-sectional	From November 2014 to November 2016.	Baseline 8,802, 53% f, 38 yr	E-cycling, days per month	Unclear	Self-perceived health
			Follow-up 3,567, 53% f, 41 yr			
	Seven European cities					
de Haas et al. (21)	Observational longitudinal study, The Netherlands	2017–2019	1,420 52% f, adults	E-cycling	Passive transport	Overweight obesity
						Self-perceived health

BMI, body mass index; f, female; m, male; SBP, systolic blood pressure; DBP, diastolic blood pressure; yr, year.

### Risk of bias

The randomized controlled trial (13) was considered to have low risk of bias while the five quasi experimental studies (15–19) and both observational studies were considered to have high risk of bias arising from the lack of randomization process. However, the RCT was analyzed with a hypothetical control group (no cycling) when included in the meta-analysis, because the control group included in the study did conventional cycling, and the RCT therefore was considered as high risk of bias in the meta-analysis. In **Table 3**, we present SMD from the RCT only and there the study has low risk of bias. None of the included studies indicated if the study protocol was pre-registered or not.

### Meta-analysis of the effect of E-cycling on health

**Figure 2:**
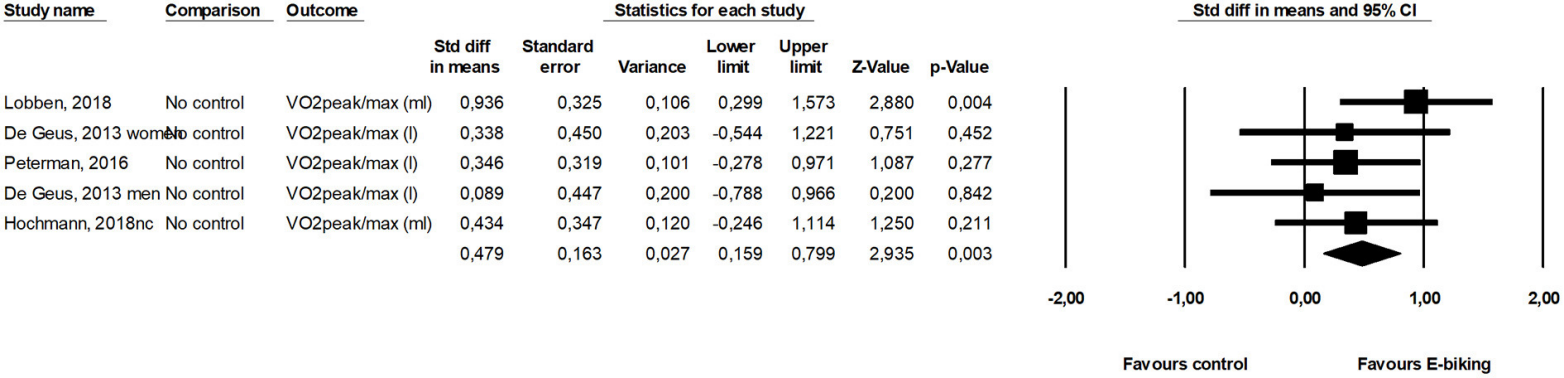
Forest plot for the effect of E-cycling on maximal oxygen consumption.

**Figure 3:**
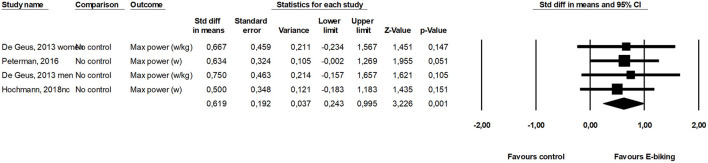
Forest plot for the effect of E-cycling on maximal power output during cycling.

Six health related outcomes were assessed in two or more included studies and were meta-analyzed. Maximal oxygen consumption (Figure 2) and maximal power output (Figure 3) increased from before to after a period with commuting with E-bike. Body mass index and blood pressure remained unchanged (Table 2). The SMD of the scores from the questionnaires assessing health also remained unchanged. The effects of the two included studies were heterogeneous.

**Table 2:** Meta-analysis for the effect of E-cycling on health outcomes.

**Outcome**	**Studies/subjects**	**Meta-analysis**			**Test of heterogeneity**	
		**SMD**	**95%CI**	***P*** **value**	**I^**2**^ (%)**	***p***
VO_2_peak/max	4/78	0.48	0.16 to 0.80	0.003	0	0.554
Max power	3/57	0.62	0.24 to 0.99	0.001	0	0.976
Physical health	2/85	0.70	−0.41 to 1.81	0.216	82	0.021
BMI	2/37	−0.06	−0.51 to 0.40	0.802	0	0.858
DBP	2/37	−0.06	−0.52 to 0.40	0.783	0	0.913
SBP	2/37	−0.12	−0.57 to 0.34	0.903	0	0.605

VO_2_peak/max, maximal oxygen consumption; Max power, maximal power output during an incremental endurance trial; BMI, body mass index; DBP, diastolic blood pressure; SBP, systolic blood pressure; SMD, standardized mean difference.

The health outcomes total cholesterol, low density lipoprotein (LDL), high density lipoprotein (HDL), triglycerides, fasting plasma glucose and 2-h post plasma glucose was assessed by Peterman et al. (19) and they reported a decrease (*p* < 0.05) in 2-h post plasma glucoses from 5.53 ± 1.18 to 5.03 ± 0.91 mmol L^−1^.

### The effect of E-cycling compared to conventional cycling

Two studies (13, 15) compared the effect of E-cycling to conventional cycling. These studies found no difference in VO_2 − max_, maximal power, mental health, BMI, DBP and SBP (*p* > 0.243) (Table 3).

**Table 3:** The effect of E-cycling compared to conventional cycling.

**Outcome**	**Subjects, E-bike/bike**	**SMD**	**95%CI**	***p***
VO_2_-peak/max	17/15	0.19	−0.54 to 0.91	0.611
Max power	17/15	0.00	−0.72 to 0.72	1.00
Mental health	38/36	0.27	−0.19 to 0.73	0.244
BMI	17/15	−0.08	−0.80 to 0.65	0.838
DBP	17/15	0.34	−0.39 to 1.07	0.356
SBP	17/15	0.20	−0.52 to 0.93	0.581

All outcomes are assessed in one study (13) only. A positive SMD would indicate positive change in favor of E-bike.

VO_2_peak/max, maximal oxygen consumption; Max power, maximal power output during an incremental endurance trial; BMI, body mass index; DBP, diastolic blood pressure; SBP, systolic blood pressure; SMD, standardized mean difference.

### Results from longitudinal observational studies

de Haas et al. (21) reported that obese people perform 0.21 times more E-bike trips than normal weight people. Obesity was not associated with E-bike distance in the same study. Self-perceived health (“How would you rate your health in general?”), or overweight was not associated with E-cycling (20, 21).

## Discussion

The aim of the present systematic review with meta-analysis was to assess the effect of E-cycling on health. We included six experimental studies with a longitudinal design (13, 15–19) and two longitudinal observational studies (20, 21). We found that regularly E-cycling improved aerobic fitness, which is an important predictor for health. There was no evidence for change in perceived physical health, BMI, systolic blood pressure or diastolic blood pressure when all available evidence was aggregated.

The meta-analysis includes only experimental trials which is considered to produce high quality evidence. The gold standard for experimental trials is randomized controlled trials and we used a quality assessment tool developed for randomized controlled trials. However, only one of our trials were properly randomized and controlled. Thus, five out of six trials were categorized as having a high risk of bias. The trial with low risk of bias compared the effect of E-cycling to conventional cycling as the only study assessing physiological outcomes. Thus, we could not use the control group in the meta-analysis which introduced a high risk of bias in this study as well.

The most studied outcomes were maximal oxygen consumption (VO_2_-max) and maximal power output. Using an E-bike increased these parameters 0.48 SD and 0.62 SD, respectively. This translates into an increase of around 10% in aerobic performance or 3.5 ml O_2_ min^−1^ kg^−1^. An increase of this size will improve health and Kodama et al. (24) found a decrease in all-cause mortality of 13% for this increase in fitness. Intuitively we would expect conventional cycling was performed at a higher intensity than E-cycling and therefore would result in greater improvements in fitness and general health. However, we speculate that when people commute, they choose a self-selected speed where they feel comfortable, because most do not want to get to work sweaty. This may apply to both conventional and E-cycling which may explain why the relative workload is almost the same. Bourne et al. (12) reported in their review similar relative intensity during E-cycling and conventional biking. The oxygen consumption ranged between 51 and 73% VO_2_-max for E-cycling and 58–74% of VO_2_-max for E-cycling conventional cycling. Nordengen et al. (1) found in a meta-analysis a 0.28 SMD in cardio respiratory fitness comparing conventional cyclists with non-cyclists, and if E-cycling provides physical activity with similar intensity it supports the findings from the present study, demonstrating improved fitness from E-cycling. Similarly, Møller et al. (2011) found an increase in VO_2_-max of 2.6 ml O_2_/min/kg (0.5 SMD) between conventional cycling and a control group in a randomized trial (25).

Other health outcomes such as blood pressure (13, 19), BMI (13, 19) and self-reported physical health (15, 16) were only assessed in two trials and total cholesterol, LDL, HDL, triglycerides, fasting plasma glucose and 2-h post plasma glucose were only assessed in one study (19). It is therefore premature to conclude the effect of E-cycling on these outcomes. Different aspects of mental and perceived health were measured in both the experimental (15, 16) and observational studies (20, 21) included in the present systematic review. However, the included studies did not find any association between mental or perceived health, and E-cycling. Previous experimental (26) and observational (20) studies have reported conventional cycling to be positively associated with mental and perceived health (20). Thus, it may seem plausible that E-cycling is associated with mental and perceived health. Our data has several weaknesses and it is therefore premature to conclude on these outcomes as well.

The strength of this review is a quantification of health effects of E-cycling in longitudinal studies. However, only one study was a randomized trial and they used conventional cycling as control. We analyzed uncontrolled longitudinal studies with a fictive control group with no change. Results should therefore be interpreted with caution. It is a weakness that all included studies had high risk of bias, and we would highly recommend future studies should be conducted as randomized controlled studies. The included studies have large variations in intervention period, type of E-bike and amount of cycling leading to great variation in exposure/physical activity. There is a dose-response relationship between physical activity and health outcomes where more physical activity is associated with better heath (7). This dose response relationship may explain why the included studies have different effect size. Another weakness is that we used RoB2, a tool intended for RCTs even if studies we identified and included were mainly not RCTs. However, the first domain in the tool “Was the allocation sequence random?” was appropriate to identify that all studies had a high risk of bias. The study included only peer reviewed studies published in English, thus there is a possibility that we have failed to include relevant studies. Still, data points against an improvement in aerobic fitness from E-cycling, and the size of the improvement is sufficient to improve health.

During the last decade sales of E-bikes has increased substantially (4) as has the use of E-bikes (19). Commuting by E-bike is a mode of active transportation providing everyday physical activity. Commuting by E-bike has a potential to improve health and we think it is surprising that there are so few longitudinal studies and only one randomized trial which investigate health effects of E-cycling. Thus, we recommend that there should be conducted more RCTs with a control group and proper randomisations investigating the effect of E-cycling over weeks or months. All health-related outcomes are of interest as long as they are assessed with valid and sensitive methods. Questions related to E-cycling should also be included in cohorts investigating health outcomes in different populations making ground for longitudinal observational studies investigating the relationships between E-cycling and health.

## Data availability statement

The original contributions presented in the study are included in the article/Supplementary material, further inquiries can be directed to the corresponding author.

## Author contributions

AR drafted the methods and results section, extracted data, performed the analysis, and finalized the manuscript. SN wrote the protocol, designed the literature search, identified eligible studies (with LA), and approved the final manuscript. LA identified eligible studies (with SN), assessed risk of bias (with EB) wrote the discussion, and approved the final manuscript. EB assessed risk of bias (with LA) wrote the introduction and approved the final manuscript. All authors contributed to the article and approved the submitted version.

## Conflict of interest

The authors declare that the research was conducted in the absence of any commercial or financial relationships that could be construed as a potential conflict of interest.

## Publisher's note

All claims expressed in this article are solely those of the authors and do not necessarily represent those of their affiliated organizations, or those of the publisher, the editors and the reviewers. Any product that may be evaluated in this article, or claim that may be made by its manufacturer, is not guaranteed or endorsed by the publisher.
